# Step Into the Examiner's Shoes: Developing a Video-Based Psychiatry Objective Structured Clinical Examination(OSCE) Revision Resource for Medical Students

**DOI:** 10.1192/bjo.2026.11329

**Published:** 2026-06-30

**Authors:** Nachammai Chockalingam, Sharna Bennett, Tiago Gameiro-Inacio, Joanne Rodda

**Affiliations:** 1 Kent and Medway Mental Health NHS Trust, Canterbury, United Kingdom; 2 Kent and Medway Medical School, Canterbury, United Kingdom; 3 Kent and Medway Mental Health NHS Trust, Kent, United Kingdom

## Abstract

**Aims:**

Objective structured clinical examination (OSCE) is a key method used widely in undergraduate medical education since 1975 to assess clinical competencies. While OSCEs have relatively high relatability, validity and objectivity, evidence shows that OSCEs are amongst the most anxiety provoking assessments. In psychiatry OSCEs, medical students often report limited understanding of examiner expectation of a competent performance, as an anxiety trigger.

Providing opportunities to medical students to assess OSCEs from an examiner perspective could help clarify expected performance standard, improve confidence and reduce OSCE-related stress.

The aim of this project was to develop video-based psychiatry OSCEs with accompanying marking rubric as a revision resource, that can be used by medical students to learn by observing and assessing performances from an examiner’s perspective.

**Methods:**

Commonly examined psychiatry scenarios at undergraduate level were selected. Candidate instructions and actor briefs were written for each selected scenario.

OSCE stations were filmed using trained actors as patients and resident doctors at various grades of training who volunteered as candidates. Candidates performed unscripted consultations after reading the candidate instructions, replicating real OSCE conditions.

For each scenario, a structured marking rubric was developed. It was specifically designed for medical students who will use it to assess the OSCE candidates while observing the recorded performances. Whilst not identical to the official examination marking scheme, the marking rubric highlights specific skills and competencies students are assessed on for each scenario and the common pitfalls.

All scenarios, candidate instructions, actors' briefs, marking rubrics and videos that were created, underwent multiple rounds of peer review to ensure accuracy, user friendliness, educational value and alignment with Kent and Medway Medical School(KMMS) examination standards.

**Results:**

Ten psychiatry OSCE station videos with corresponding marking rubrics have been created. The marking rubrics are designed to facilitate self-directed learning with ease ofstudent use in mind. This resource offers a flexible, repeatable approach to OSCE preparation.

**Conclusion:**

This collection of psychiatry OSCE videos and accompanying marking rubrics, have the potential to reduce assessment-related anxiety and improve OSCE performance. Furthermore, the added advantage of this resource is that it will be accessible to medical students who can utilise it at their own pace from any location.

Next steps include piloting the resource with third year KMMS medical students to evaluate educational impact.

